# Author Correction: Membrane translocation process revealed by in situ structures of type II secretion system secretins

**DOI:** 10.1038/s41467-023-40656-5

**Published:** 2023-08-10

**Authors:** Zhili Yu, Yaoming Wu, Muyuan Chen, Tong Huo, Wei Zheng, Steven J. Ludtke, Xiaodong Shi, Zhao Wang

**Affiliations:** 1 Verna and Marrs McLean Department of Biochemistry and Molecular Biology, Baylor College of Medicine, Houston, TX 77030 USA; 2 Jiangsu Province Key Laboratory of Anesthesiology and Jiangsu Province Key Laboratory of Anesthesia and Analgesia Application, Xuzhou Medical University, Xuzhou, Jiangsu 221004 China; 3 Cryo Electron Microscopy and Tomography Core, Baylor College of Medicine, Houston, TX 77030 USA; 4 Department of Molecular and Cellular Biology, Baylor College of Medicine, Houston, TX 77030 USA; 5 Present Address: Division of CryoEM and Bioimaging, SSRL, SLAC National Accelerator Laboratory, Stanford University, Menlo Park, CA 94025 USA

**Keywords:** Cryoelectron tomography, Bacterial secretion, Structural biology

Correction to: *Nature Communications* 10.1038/s41467-023-39583-2, published online 07 July 2023

The original version of this Article contained an error the acknowledgements section, which incorrectly omitted the following: “CryoEM data was collected at the Baylor College of Medicine CryoEM ATC and UTHealth cryoEM core, which includes equipment purchased under the support of CPRIT Core Facility Award RP190602. “. This has been corrected in both the PDF and HTML versions of the Article.

