# Supplementary material for: Membrane translocation process revealed by in situ structures of type II secretion system secretins
Source: Nat Commun. 2023 Jul 7;14:4025. doi: 10.1038/s41467-023-39583-2 (PMC10329019; doi:10.1038/s41467-023-39583-2)
Supplement: Supplementary file 1 — Supplementary Information [file 41467_2023_39583_MOESM1_ESM.pdf]

Membrane translocation process revealed by *in situ* structures of type II secretion system secretins

Zhili Yu[1†], Yaoming Wu[2†], Muyuan Chen[1#], Tong Huo[1], Wei Zheng[2], Steven J. Ludtke[1,3], Xiaodong Shi[2*], Zhao Wang[1,3,4*]

[1]Verna and Marrs McLean Department of Biochemistry and Molecular Biology, Baylor College of Medicine, Houston, TX, 77030, USA.

[2]Jiangsu Province Key Laboratory of Anesthesiology and Jiangsu Province Key Laboratory of Anesthesia and Analgesia Application, Xuzhou Medical University, Xuzhou, Jiangsu 221004, China.

[3]Cryo Electron Microscopy and Tomography Core, Baylor College of Medicine, Houston, TX, 77030, USA.

[4]Department of Molecular and Cellular Biology, Baylor College of Medicine, Houston, TX, 77030, USA.

[#]Present address: Division of CryoEM and Bioimaging, SSRL, SLAC National Accelerator Laboratory, Stanford University, Menlo Park, CA, 94025, USA.

[†]These authors contributed equally to this work.

*Corresponding author. Email: shixd@xzhmu.edu.cn; zhaow@bcm.edu.

Supplementary Tables

Supplementary Table 1. Statistics of *in situ* structures achieved in this study. IM, inner membrane; OM, outer membrane.

Structure	$GspD_{\alpha}$ on IM	$GspD_{\alpha}$ on OM	$GspD_{\beta}$-GspS on OM	$GspD_{\beta}$-GspS on OM	$GspD_{\beta}$ on IM
Overexpressed protein(s)	$GspD_{\alpha}$	$GspD_{\alpha}$	$GspD_{\beta}$ and GspS	$GspD_{\beta}$	$GspD_{\beta}$
Microscope	Titan Krios	Titan Krios	Glacios	Glacios	Glacios
Tilt scheme	Bidirectional	Bidirectional	Dose symmetry	Dose symmetry	Dose symmetry
Angstrom per pixel	1.76	1.76	1.52	1.52	1.52
Tilt series number	250	91	63	191	24
Particle number	32,915	309	514	723	370
Resolution (C15)	9 Å	16 Å	19 Å	16 Å	14 Å

Supplementary Figures

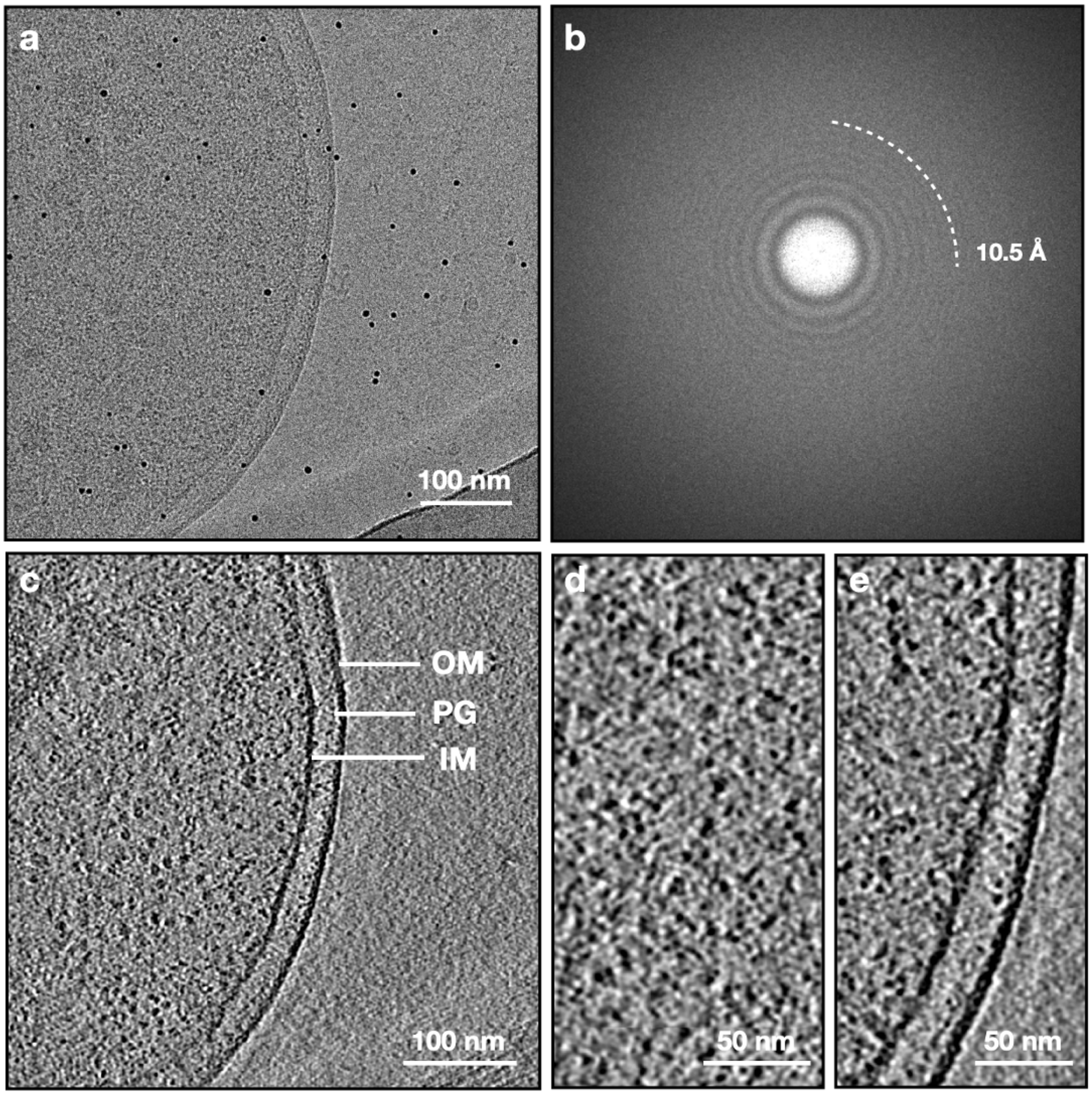

Supplementary Fig. 1 The cryo-ET tomogram of control *E. coli* minicells not overexpressing any protein. a, A tilt image at 0 degrees. b, The Fourier transform of a, showing the resolving ability. c, The reconstructed tomogram of an *E. coli* minicell not expressing any protein. d, Zoomed in tomogram z slice view of an area on the cell surface. e, Zoomed in tomogram z slice view of the cell envelope region. OM, outer membrane; IM, inner membrane.

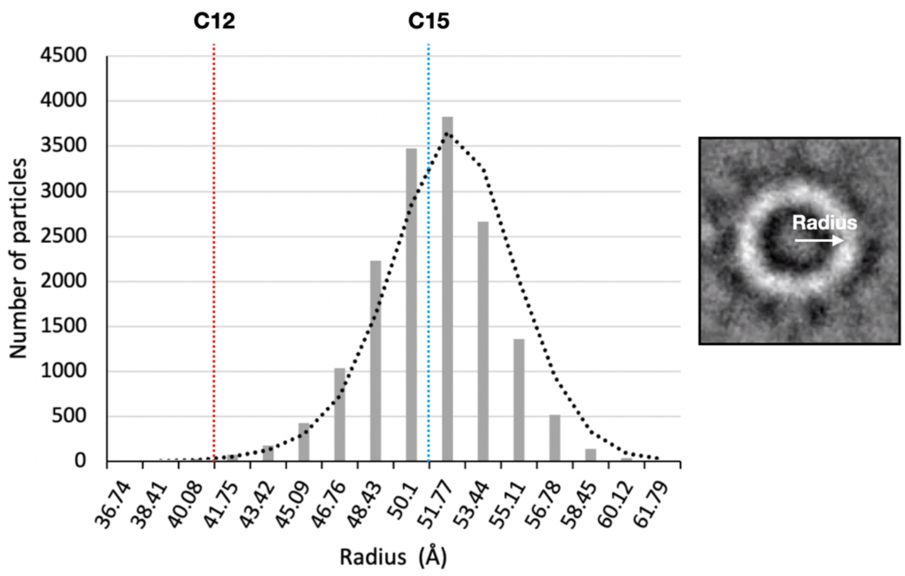

Supplementary Fig. 2 The histogram distribution of the radius of GspD$_\alpha$ particles *in situ*. The trend line is the moving average of the histogram data. A particle top view 2D image is shown on the right to illustrate the way of measuring the radius. The C15 particle radius is indicated by the blue dashed line, the radius measured from 5WQ7 [https://doi.org/10.2210/pdb5WQ7/pdb] as the average radius of the inner and outer β-barrels. The putative C12 particle radius is indicated by the red dashed line, calculated from the C15 particle radius, assuming each monomer occupies the same length of the circumference. Source data are provided as a Source Data file.

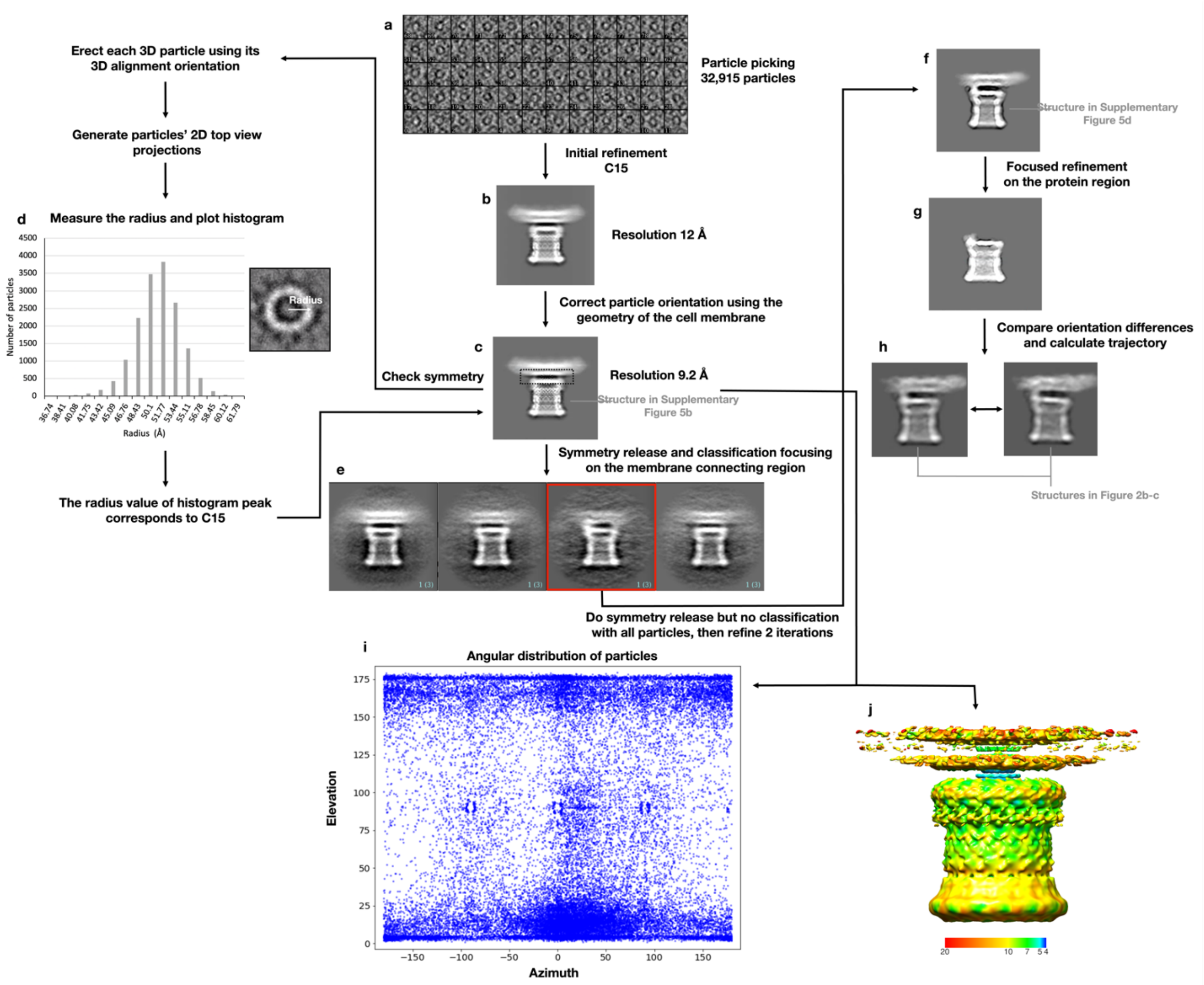

Supplementary Fig. 3 The data processing workflow for the *E. coli* overexpressing GspD$_{\alpha}$ dataset. After the manual boxing of particles (a), an initial refinement was done with C15 symmetry (b). Then the cell membrane feature was used to correct the particle orientations (see Methods). A local refinement was performed with the corrected orientation as input, achieving the 9.2 Å resolution structure (c). To verify the symmetry here, we measured and plotted the particle radius (d). The symmetry release and classification were focused on the membrane connecting region marked by the dashed line box in c, which resulted in four classes (e), including a class that shows an obvious uneven connection to the membrane (red line box in e). We then did a symmetry release with the whole dataset, followed by 2 iterations of refinement, producing the C1 structure

(f). To demonstrate the particle movement, we performed a focused refinement with a mask only covering the protein density (g) and compared the orientation difference to that from the integral refinement. Conformations within this movement could be presented by calculating the trajectory and interval class averages on the trajectory (h). The angular distribution of particles used in the final reconstruction is shown in (i), with the *y*-axis being the orientation of the symmetry axis (elevation), and the *x*-axis being the angle of the particle around the symmetry axis (azimuth). In the final local orientation search, the range of the angle is not limited by the symmetry imposed and the particle orientation can be assigned to neighboring asymmetrical units during the refinement. The map colored to local resolution is shown in (j).

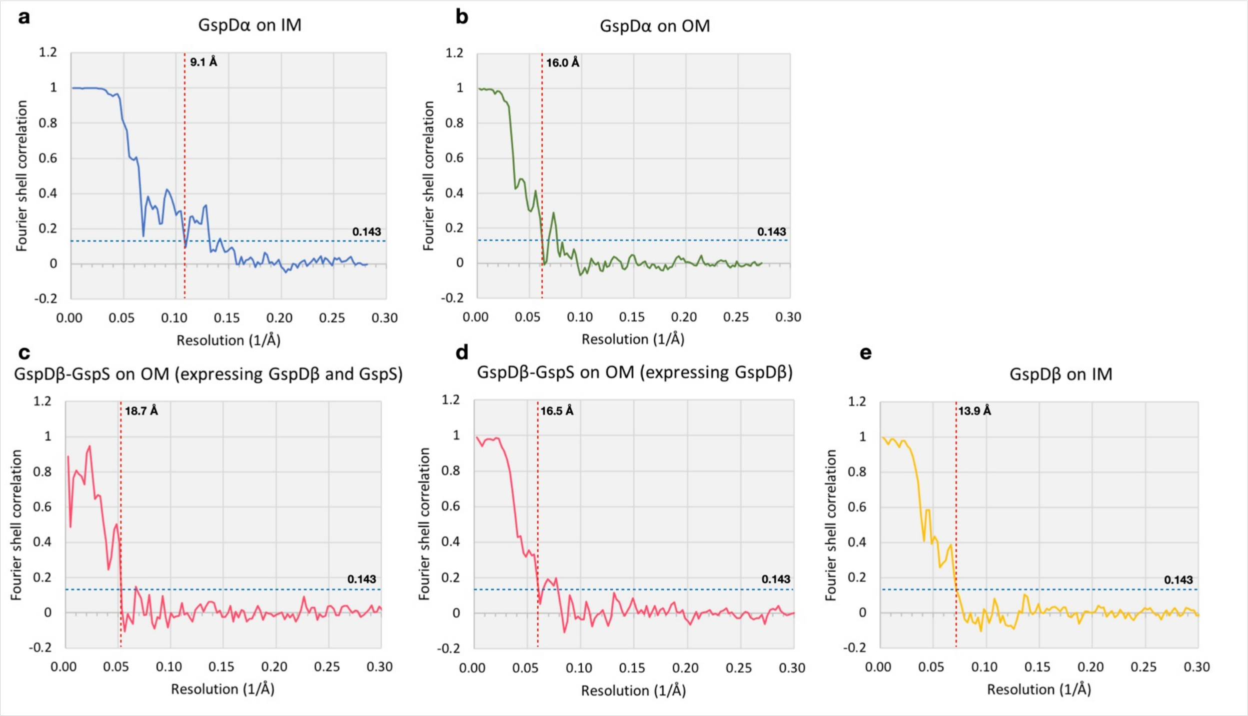

Supplementary Fig. 4 The FSC curves showing the resolution of *in situ* structures achieved in this work. Source data are provided as a Source Data file.

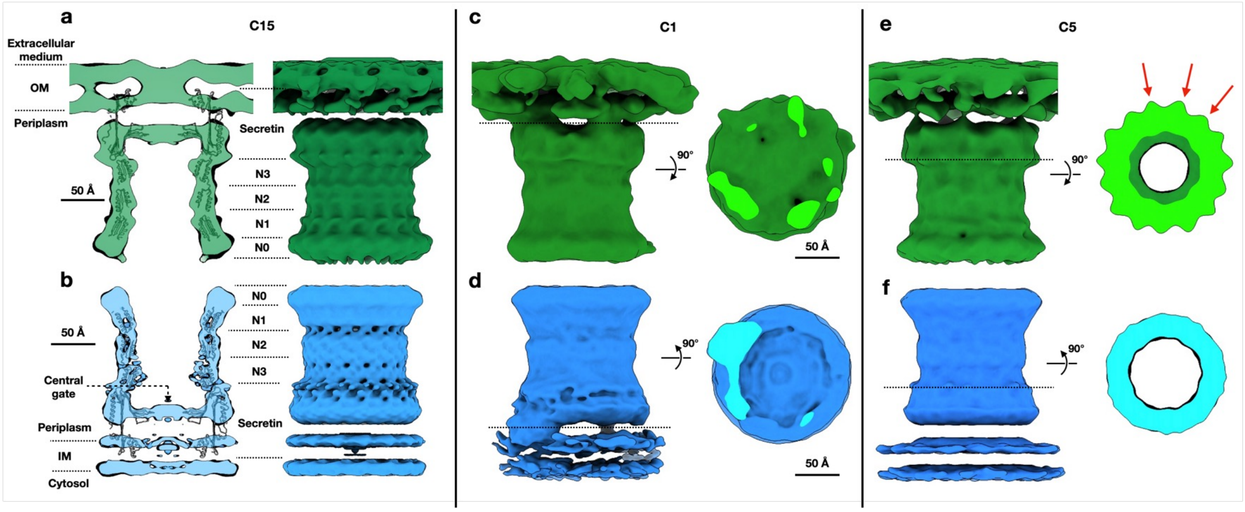

Supplementary Fig. 5 Comparison of GspD$_\alpha$ *in situ* structures on the inner and outer membranes. a, The *in situ* structure of GspD$_\alpha$ on the outer membrane, applied with C15 symmetry, fitted with GspD$_\alpha$ *in vitro* structure (PDB: 5WQ7 [https://doi.org/10.2210/pdb5WQ7/pdb]). b, The *in situ* structure of GspD$_\alpha$ on the inner membrane, applied with C15 symmetry, fitted with GspD$_\alpha$ *in vitro* structure (PDB: 5WQ7 [https://doi.org/10.2210/pdb5WQ7/pdb]). c, The *in situ* structure of GspD$_\alpha$ on the outer membrane, symmetry released. d, The *in situ* structure of GspD$_\alpha$ on the inner membrane, symmetry released. e, The *in situ* structure of GspD$_\alpha$ on the outer membrane, applied with C5 symmetry. The three units within one C5 symmetry unit are indicated by red arrows. f, The *in situ* structure of GspD$_\alpha$ on the inner membrane, applied with C5 symmetry. IM, inner membrane; OM, outer membrane.

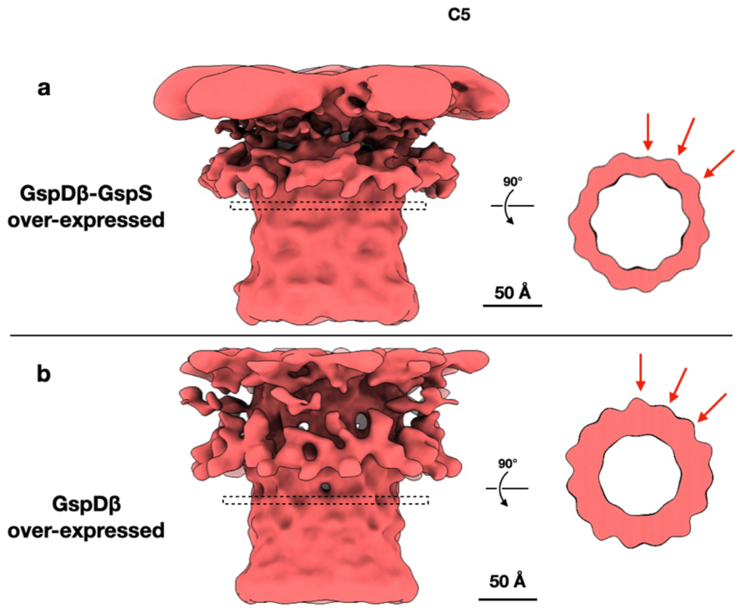

Supplementary Fig. 6 The *in situ* structures of the GspD$_{\beta}$-GspS complex with C5 symmetry applied. a, Both GspD$_{\beta}$ and GspS are overexpressed; b, Only GspD$_{\beta}$ is overexpressed. The cross-section at the position indicated by the dashed box is shown on the right. The three units within one C5 symmetry unit are indicated by red arrows.

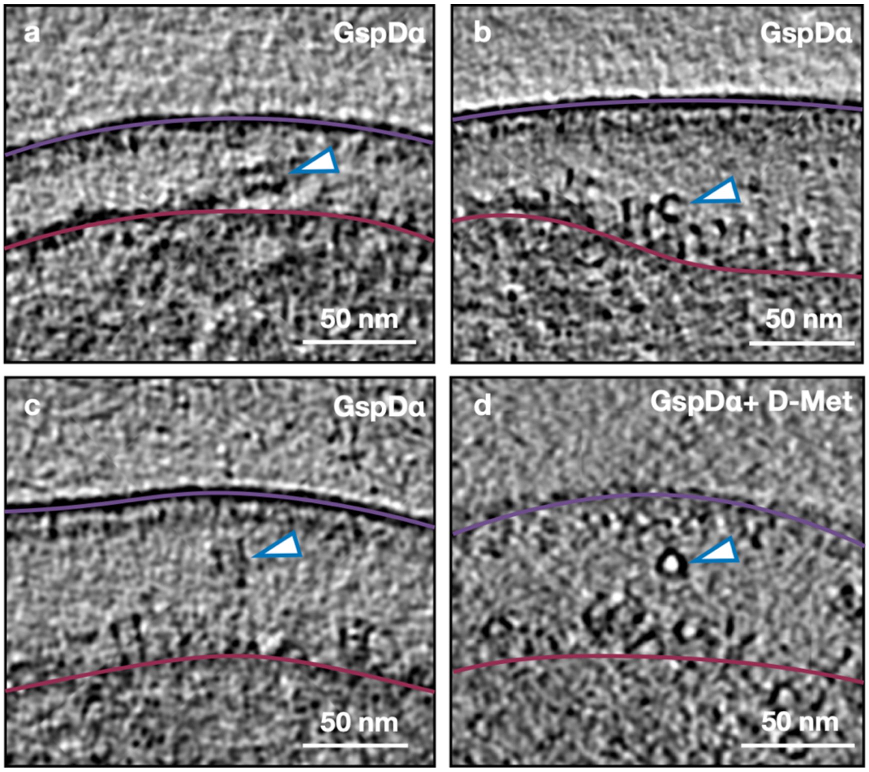

Supplementary Fig. 7 GspD$_\alpha$ multimer particles in the periplasm. a, b, and c, Tomogram z slice view of the *E. coli* cells overexpressing GspD$_\alpha$. d, Tomogram z slice view of an *E. coli* cell overexpressing GspD$_\alpha$ and with D-methionine added. The outer and inner membranes are indicated by the purple line and the dark-red line, respectively. The particles inside the periplasm are indicated by white arrowheads with blue outlines. D-Met, D-methionine.

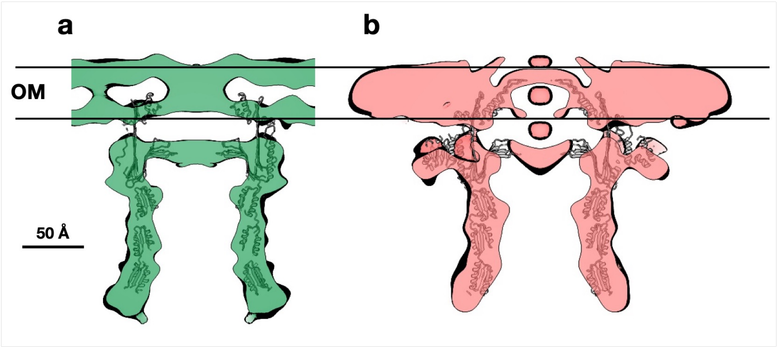

Supplementary Fig. 8 Comparison of the transmembrane regions of GspD$_{\alpha}$ and GspD$_{\beta}$ *in situ* structures on the outer membrane. a, GspD$_{\alpha}$ on the outer membrane, fitted by 5WQ7 [https://doi.org/10.2210/pdb5WQ7/pdb]. b, GspD$_{\beta}$ on the outer membrane, fitted by 5ZDH [https://doi.org/10.2210/pdb5ZDH/pdb]. OM, outer membrane**.**
