# Supplementary material for: Membrane translocation process revealed by in situ structures of type II secretion system secretins
Source: Nat Commun. 2023 Jul 7;14:4025. doi: 10.1038/s41467-023-39583-2 (PMC10329019; doi:10.1038/s41467-023-39583-2)
Supplement: Supplementary file 3 — Description of Additional Supplementary Files [file 41467_2023_39583_MOESM3_ESM.pdf]

Description of Additional Supplementary Files

Movie Legends

Supplementary Movie 1. Visualization of GspDα on the E. coli inner membrane. IM, inner membrane; OM, outer membrane; PG, peptidoglycan.

Supplementary Movie 2. The symmetry release of GspDα and the movement of GspDα on the inner membrane. IM, inner membrane.
