# Supplementary material for: Membrane translocation process revealed by in situ structures of type II secretion system secretins
Source: Nat Commun. 2023 Jul 7;14:4025. doi: 10.1038/s41467-023-39583-2 (PMC10329019; doi:10.1038/s41467-023-39583-2)
Supplement: Supplementary file 6 — Reporting Summary [file 41467_2023_39583_MOESM6_ESM.pdf]

nature portfolio

Corresponding author(s): Zhao Wang, Xiaodong Shi

Last updated by author(s): Jun 14, 2023

# Reporting Summary

Nature Portfolio wishes to improve the reproducibility of the work that we publish. This form provides structure for consistency and transparency in reporting. For further information on Nature Portfolio policies, see our Editorial Policies and the Editorial Policy Checklist.

## Statistics

For all statistical analyses, confirm that the following items are present in the figure legend, table legend, main text, or Methods section.

| n/a | Confirmed | |
|---|---|---|
| ☒ | ☐ | The exact sample size ($n$) for each experimental group/condition, given as a discrete number and unit of measurement |
| ☒ | ☐ | A statement on whether measurements were taken from distinct samples or whether the same sample was measured repeatedly |
| ☒ | ☐ | The statistical test(s) used AND whether they are one- or two-sided<br>*Only common tests should be described solely by name; describe more complex techniques in the Methods section.* |
| ☒ | ☐ | A description of all covariates tested |
| ☒ | ☐ | A description of any assumptions or corrections, such as tests of normality and adjustment for multiple comparisons |
| ☒ | ☐ | A full description of the statistical parameters including central tendency (e.g. means) or other basic estimates (e.g. regression coefficient) AND variation (e.g. standard deviation) or associated estimates of uncertainty (e.g. confidence intervals) |
| ☒ | ☐ | For null hypothesis testing, the test statistic (e.g. $F$, $t$, $r$) with confidence intervals, effect sizes, degrees of freedom and $P$ value noted<br>*Give P values as exact values whenever suitable.* |
| ☒ | ☐ | For Bayesian analysis, information on the choice of priors and Markov chain Monte Carlo settings |
| ☒ | ☐ | For hierarchical and complex designs, identification of the appropriate level for tests and full reporting of outcomes |
| ☒ | ☐ | Estimates of effect sizes (e.g. Cohen's $d$, Pearson's $r$), indicating how they were calculated |

*Our web collection on statistics for biologists contains articles on many of the points above.*

## Software and code

Policy information about availability of computer code

| | |
|---|---|
| Data collection | SerialEM version 3.8, Tomography version 5 |
| Data analysis | EMAN2 version 2.91, MotionCor2 version 1.1.0 |

For manuscripts utilizing custom algorithms or software that are central to the research but not yet described in published literature, software must be made available to editors and reviewers. We strongly encourage code deposition in a community repository (e.g. GitHub). See the Nature Portfolio guidelines for submitting code & software for further information.

## Data

Policy information about availability of data

All manuscripts must include a data availability statement. This statement should provide the following information, where applicable:

- Accession codes, unique identifiers, or web links for publicly available datasets
- A description of any restrictions on data availability
- For clinical datasets or third party data, please ensure that the statement adheres to our policy

Density maps are deposited in EMDB with accession codes: EMD-29702 [https://www.ebi.ac.uk/emdb/EMD-29702] (GspDα on the inner membrane), EMD-29703 [https://www.ebi.ac.uk/emdb/EMD-29703] (GspDα on the outer membrane), EMD-29698 [https://www.ebi.ac.uk/emdb/EMD-29698] (GspDβ on the inner membrane), EMD-29697 [https://www.ebi.ac.uk/emdb/EMD-29697] (GspDβ-GspS on the outer membrane, expressing only GspDβ), and EMD-29696 [https://

www.ebi.ac.uk/emdb/EMD-29696] (GspDβ-GspS on the outer membrane, expressing GspDβ and GspS).

## Human research participants

Policy information about studies involving human research participants and Sex and Gender in Research.

| | |
|---|---|
| Reporting on sex and gender | Not applicable. |
| Population characteristics | Not applicable |
| Recruitment | Not applicable |
| Ethics oversight | Not applicable |

Note that full information on the approval of the study protocol must also be provided in the manuscript.

# Field-specific reporting

Please select the one below that is the best fit for your research. If you are not sure, read the appropriate sections before making your selection.

☒ Life sciences ☐ Behavioural & social sciences ☐ Ecological, evolutionary & environmental sciences

For a reference copy of the document with all sections, see nature.com/documents/nr-reporting-summary-flat.pdf

# Life sciences study design

All studies must disclose on these points even when the disclosure is negative.

| | |
|---|---|
| Sample size | Since our sample is not statistical sample, and we are not making inferences about a population based on a sampling, the sample size is not applicable. |
| Data exclusions | No data were excluded from the analyses. |
| Replication | All attempts at replication were successful. |
| Randomization | Since our research does not contain the process of assigning individuals to different groups with different treatment, the randomization is not applicable. |
| Blinding | Since our research does not contain the process of assigning individuals to different groups with different treatment, the blinding is not applicable. |

# Reporting for specific materials, systems and methods

We require information from authors about some types of materials, experimental systems and methods used in many studies. Here, indicate whether each material, system or method listed is relevant to your study. If you are not sure if a list item applies to your research, read the appropriate section before selecting a response.

### Materials & experimental systems

| n/a | Involved in the study | |
|---|---|---|
| ☐ | ☒ | Antibodies |
| ☒ | ☐ | Eukaryotic cell lines |
| ☒ | ☐ | Palaeontology and archaeology |
| ☒ | ☐ | Animals and other organisms |
| ☒ | ☐ | Clinical data |
| ☒ | ☐ | Dual use research of concern |

### Methods

| n/a | Involved in the study | |
|---|---|---|
| ☒ | ☐ | ChIP-seq |
| ☒ | ☐ | Flow cytometry |
| ☒ | ☐ | MRI-based neuroimaging |

## Antibodies

| | |
|---|---|
| Antibodies used | HRP-conjugated 6*His, His-tag mouse monoclonal antibody. Supplier: proteintech. Catalog number: HRP-66005. Clone Number: 1B7G5. |

Validation

Recombinant protein were subjected to SDS PAGE followed by western blot with HRP-66005 (6*His, His-Tag antibody) at dilution of 1:10000 incubated at room temperature for 1.5 hours. Application: western blot. Positive western blot detected in recombinant protein.
